# Gastrojejunostomy for Pyloric Obstruction1Read at the 36th annual meeting of the Medical Association of Central New York, held at Auburn, September 22, 1903.

**Published:** 1903-11

**Authors:** William S. Cheesman

**Affiliations:** Auburn, N.Y.


### Gastrojejunostomy for Pyloric Obstruction.[1]

BY WILLIAM S. CHEESMAN, M. D., Auburn, N. Y.

THE patient, a tall emaciated farmer, aged 56, consulted me December 31, 1902. Thirty years ago he was laid up a month with pain in the region of the stomach, but so far as he knew, did not vomit or pass blood. Ever since then he has been subject to attacks of pain. These became so constant that he needed morphine daily by hypodermic. He vomited large quantities about once a week, but never blood. No tumor was palpable in the abdomen, but gastrectasia was demonstrated by ausculatory percussion and inflation. There was marked atheroma of the larger arteries, but the urine contained no albumin or sugar.

At the Auburn City Hospital a test meal showed presence of hydrochloric and lactic acids. Gastric lavage was practised here daily for a month, and the patient fed only peptonised milk and eggs. The relief from pain was immediate, morphine was stopped, and some gain in weight ensued, but it was soon apparent that the limit of improvement had been reached, and operation was resorted to on February 11, 1903. The diagnosis before admission had favored cancer of the stomach. This, however, seemed ruled out by the long history, the absence of palpable growth, and the presence of hydrochloric acid in the gastric contents. Hence, cicatricial, nonmalignant obstruction of the pylorus, dating from ulcer thirty years ago, was diagnosticated, and the operation planned was either a Heinike-Mikulicz plastic on the pylorus, or a gastrojejunostomy.

Just before giving the anesthetic thorough washing of the stomach was practised, and when as much of the irrigating fluid returned as would, the patient was elevated to the Trendelenburg position, and the tube gradually withdrawn. This thoroughly emptied the stomach, as was shown later when the organ was opened. Dr. Neck (*Centralblatt für Chir.*, December 27, 1902, "Zur Technik der Magenausspulung") has called attention to the value of the maneuver. With the patient upside-down the residuum of the lavage fluid gravitates to the top of the cardiac cone, and, as the tube recedes, flows out to the last drop. This is an important achievement, as we thus avoid: first, embarrassment and possible infection from fluid flowing out over the field

---

1. Read at the 36th annual meeting of the Medical Association of Central New York, held at Auburn, September 22, 1903.

when the stomach is opened; second, the danger of self-strangulation by inhalation of vomited fluids; third, when the stomach is lifted during the manipulations any fluid it contains may gravitate up the esophagus and into the trachea,—not a fanciful peril by any means, as Dr. W. J. Mayo reports one death from this cause.

Under chloroform an incision was made from the ensiform cartilage to the umbilicus in the middle line. On opening the abdomen a hard cicatricial mass was felt at the pylorus, from which dense adhesions radiated to the structures about. The pylorus was thereby immobilised, and delivery of the mass through the wound prevented. The plastic operation of Heinike-Mikulicz was therefore rejected. It may be observed in passing that the usefulness of the ingenious device of Heinike-Mikulicz is now regarded as very limited. Unless the pylorus is movable, permitting the stomach to take a nearly perpendicular position, the organ is not drained satisfactorily, no matter how large a pyloric opening be made. Adhesions, if not present before, may form after operation, immobilising the pylorus in a high position, when the degenerated musculature of a chronically dilated stomach is unable to lift its contents to the opening (Mayo). Of 19 cases of pyloroplasty done by Mayo, 6 came to secondary gastrojejunostomy owing to failure to drain the stomach.

Accordingly, recourse was had to gastrojejunostomy. Again, referring to the experience of Mayo, which is probably greater than that of any other operator, it matters little whether the anterior or posterior anastomosis be made, provided the lowest point of the greater curvature be selected. I did the posterior operation, omentum, transverse colon, and stomach being held upward, and a hole torn in the mesocolon admitting to the cavity of the lesser omentum and the posterior wall of the stomach. The jejunum was then traced to its emergence beneath the ligament of Treitz, and stomach and gut united by a running suture of fine silk for 2½ inches, a slit cut in stomach and gut, the two edges whipped with catgut, and the first mentioned silk suture completed around the front. This suture of the raw edges of the anastomotic opening must be thoroughly done, else the mucous membrane may adhere from side to side, permanently closing the opening. W. H. Brown (*Lancet,* July 7, 1901), reports a gastrojejunostomy which did well for two weeks, when obstruction recurred. On secondary operation, no cause being discovered outside, the stomach was reopened, when it was found that a membrane had formed across the anastomotic aperture. This was torn through, the stomach closed, and recovery followed.

The next and last step was a jejunojejunostomy by Murphy button. There has been discussion as to the need of this additional opening, and doubtless some cases have recovered without it. Its purpose is to prevent the so-called "vicious circle", a reflux of digested matters from the stomach into the afferent loop of the duodenum, thence back into the stomach, and so on, over and over, leading to uncontrollable vomiting, exhaustion, and death. Probably dilatation of the duodenum from imperfect emptying of bilious, pancreatic, and other fluids also plays a rôle in this condition. But that the entry of bile into the stomach is the main element in causation, is denied for the reason that experimental anastomoses (Masse and Terrier), between gall-bladder or common duct and stomach, compelling the whole biliary secretion to traverse that organ, have not been followed by vicious circle.

That an opening between the two limbs of the attached jejunum is usually needful is evidenced by the experience of F. Franke (*Centralblatt f. Chir*, No. 14, 1901), who lost a case by "circulus vitiosus", and found at autopsy that the enteroanastomosis had closed, the mucous membrane, which had not been included in the suture, having united across the opening, another illustration of the need of thorough suturing already mentioned. Mikulicz saved four out of eight cases of bilious vomiting by reopening and doing an enteroanastomosis, and Mayo had in three cases to do a secondary enteroanastomosis to relieve vicious circle. It is, therefore, doubtless wise to do this at the first sitting.

During the operation the patient breathed badly, but went to bed in good condition. Was nourished by rectum till fourth day, when peptonised milk was begun. Eggs were added on the seventh day. The appetite became enormous and virtually insatiable, and weight was put on rapidly. He returned to his home five weeks after operation, the Murphy button having never been found in the passages. He now weighs 165 pounds, eats a generous and varied diet, and has done farm work, plowing, haying, and the like, all summer.

Not many advances have been made in gastric surgery that have not been noticed in the exhaustive articles by Weir, Keen, Frazier, and Bevan. (Weir: on the operation of gastroenterostomy, combined with enteroanastomosis, *N. Y. Med Record,* April 16, 1898. W. W. Keen: Cartwright lectures on surgery of the stomach, *N. Y. Med. Jour.,* May 7, 1898. Chas. H. Frazier: surgery of the stomach, *Amer. Jour. Med. Sci.,* May, 1900. A. D. Bevan: surgery of the stomach, *Jour. Amer. Med. Assoc.,* January 24, 1903).

I find only the following points to bring to your attention. In the *Annals of Surgery,* November, 1902. Fowler describes a method of his own to prevent the occurrence of "vicious circle." He encircles the afferent jejunal arm with silver wire just tightly enough to occlude, but not to cut it, thus preventing duodenal contents from entering the stomach, or vice versa. I cannot, however, discern wherein this modification differs from that proposed by Chaput, except that the latter used silk where Fowler uses wire, and tried to make his opening between stomach and bowel a valvular one.

The plan of Max Rutkowski is winning some favorable comment. (Centralblatt f. Chir., No. 39, 1899.) It consists in doing a Witzel or Kader gastrostomy, in addition to the gastroenterostomy. A tube is then introduced from outside into the stomach, and thence through the anastomosis into the efferent jejunal limb, where Witzel thinks it should be made fast with catgut. This permits immediate and continued feeding into the bowel, a great advantage in cases weakened by starvation, and it lessens postoperative vomiting. Hammesfahr (Zeitsch, f. Chir., June 6, 1903), reports 13 cases so treated with no deaths, and no vomiting following operation.

Witzel claims to have been doing this operation independently, and names it gastroenterostomosis externa.

Doubtless the most noteworthy recent addition to the technique of gastroenterostomy was that of McGraw. (Gastroenterostomy by the elastic ligature., *N. Y. Med. Jour.*, January 26, 1901). Dr. McGraw does not claim originality for the idea, which he credits to Gaston, of Atlanta, (1884), and Bardenhauer, (1888). Modlinski, of Moscow, at the sitting of the *Deutsche Gesellschaft für Chirurgie*, April 5, 1899, refers to a number of experiments by different surgeons in the use of silk ligature in effecting gastroenterostomy. Of these Podres's plan to connect stomach and bowel by two silk ligatures at right angles, attracted most notice. It was found, however, that no permanent opening followed this method, the silk embedding itself in the tissues, which healed over it as fast as it cut, till it eventually remained buried. So Modlinski proposed the rubber ligature to replace silk, his technique being in all respects similar to McGraw's, and reported nine successful experiments on dogs. But McGraw was the first to do this operation successfully on man, and since his publication the method has found wide acceptance, and in view of its simplicity, and rapidity of execution, doubtless has a brilliant future. (For excellent illustrations of the steps of McGraw's operation, see *Jour. Am. Med. Assoc.*, January 17, 1903).

*Mortality.*—In 121 gastroenterostomies for nonmalignant pyloric obstruction, Mayo reports 8 per cent. of deaths, and 30 per cent. in malignant cases. Bevan found that 190 malignant cases (combining those of Rydygier, Kronlein, Maydl, Kocher, Czerny, Morrison, Mayo, and his own), gave 50 deaths, or 26 per cent. mortality. Of the 140 recoveries, only 17 lived more than three years, and some of these with recurrences. So, only 5 per cent. were really cured.

But in benignant stenosis of the pylorus gastroenterostomy, combined with enteroenterostomy, now holds undisputed preeminence as a life saving and health restoring operation.

